# The Effect of Volatile Organic Compounds on Different Organisms: Agrobacteria, Plants and Insects

**DOI:** 10.3390/microorganisms10010069

**Published:** 2021-12-30

**Authors:** Daria E. Sidorova, Vladimir A. Plyuta, Darya A. Padiy, Evgeniya V. Kupriyanova, Natalia V. Roshina, Olga A. Koksharova, Inessa A. Khmel

**Affiliations:** 1 Institute of Molecular Genetics of National Research Center “Kurchatov Institute”, Kurchatov sq. 2, 123182 Moscow, Russia; misenok1@gmail.com (D.E.S.); plyutaba@gmail.com (V.A.P.); padydarya@gmail.com (D.A.P.); nwumr@yandex.ru (N.V.R.); koksharova@belozersky.msu.ru (O.A.K.); 2 Department of Biotechnology, Mendeleev University of Chemical Technology of Russia, 125480 Moscow, Russia; 3 Department of Genetics, Faculty of Biology, Lomonosov Moscow State University, Leninskiye Gory 1/12, 119234 Moscow, Russia; ekupriyanova@gmail.com; 4 A.N. Belozersky Institute of Physico-Chemical Biology, Lomonosov Moscow State University, Leninskie Gory 1-40, 119991 Moscow, Russia

**Keywords:** volatile organic compounds, ketones, alcohols, terpenes, *Agrobacterium tumefaciens*, *Arabidopsis thaliana*, *Drosophila melanogaster*

## Abstract

Bacteria and fungi emit a huge variety of volatile organic compounds (VOCs) that can provide a valuable arsenal for practical use. However, the biological activities and functions of the VOCs are poorly understood. This work aimed to study the action of individual VOCs on the bacteria *Agrobacterium tumefaciens*, *Arabidopsis thaliana* plants, and fruit flies *Drosophila melanogaster*. VOCs used in the work included ketones, alcohols, and terpenes. The potent inhibitory effect on the growth of *A. tumefaciens* was shown for 2-octanone and isoamyl alcohol. Terpenes (−)-limonene and (+)-α-pinene practically did not act on bacteria, even at high doses (up to 400 µmol). 2-Butanone and 2-pentanone increased the biomass of *A. thaliana* at doses of 200–400 μmol by 1.5–2 times; 2-octanone had the same effect at 10 μmol and decreased plant biomass at higher doses. Isoamyl alcohol and 2-phenylethanol suppressed plant biomass several times at doses of 50–100 μmol. Plant seed germination was most strongly suppressed by isoamyl alcohol and 2-phenylethanol. The substantial killing effect (at low doses) on *D. melanogaster* was exerted by the terpenes and the ketones 2-octanone and 2-pentanone. The obtained data showed new information about the biological activities of VOCs in relation to organisms belonging to different kingdoms.

## 1. Introduction

In recent years, volatile organic compounds (VOCs) emitted by microorganisms have attracted great interest among researchers working in microbiology, biotechnology, medicine, and agriculture. VOCs are mainly lipophilic compounds with small molecular masses (on average below 300 Da), low boiling points, and high vapor pressure. VOCs can spread through air and liquids, acting over short and long distances [1,2,3,4,5,6,7].

A database of identified VOCs (mVOC 2.0 database) has been published (http://bioinformatics.charite.de/mvoc/, accessed on 16 March 2021); it includes more than 2000 compounds emitted by about 1000 species of bacteria and fungi [8]. However, this is only a small part of volatile substances and their producers due to the difficulty of their identification and a small number of studied microbial strains.

Bacterial VOCs belong to different chemical types, including ketones, alcohols, terpenoids, sulfur-containing compounds, alkenes, etc. Some substances are common to the whole group of microorganisms, but others are specific only for particular strains. One bacterium can synthesize up to 100 different VOCs. The synthesis of microbial VOCs is a complex and multifactorial process. The composition of VOCs emitted can depend on many factors, such as the composition of the nutrient medium, pH, aeration, stage of culture growth, etc. [9,10].

It has been shown that microorganisms can play a significant role in either antagonistic or positive interactions between microorganisms and inter-kingdom communication due to these compounds. VOCs can modulate the growth and development of microorganisms and plants (inhibit or stimulate); cause systemic resistance of plants; and affect insects, nematodes, and other organisms. VOCs synthesis may be of importance in microbial competition within an ecological niche (e.g., in the rhizosphere of plants), in the antagonistic relationships between plant-pathogenic and plant-associated bacteria, microorganisms of human and animal microflora [1,7,11,12,13,14,15,16,17]. VOCs can affect the quorum-sensing (QS) cell-to-cell communication network, increasing or decreasing QS regulation (quorum sensing quenching effect, QQ) [5,18,19,20,21].

Despite the great interest of researchers in VOCs and promising prospects for their use in practice, the mechanisms of action of VOCs are poorly understood. The main attention was paid to studying the effects of the total pools of gaseous mixtures released by strains—antagonists of phytopathogenic microorganisms on plants and fungi and their role in the biocontrol of plant diseases. Furthermore, although this approach is closer to natural conditions, for a clear understanding of the patterns and mechanisms of VOC action, it is necessary to study the effects of individual pure compounds, which are little researched.

Previously, we had found out that ketones 2-nonanone, 2-undecanone, 2-heptanone, sulfur-containing compound dimethyl disulfide (DMDS), and alkene 1-undecene emitted by *Pseudomonas* and *Serratia* strains have an inhibitory and killing effect on phytopathogenic bacteria *A. tumefaciens,* insects *D. melanogaster*, nematodes *Caenorhabditis elegans* [22], and plant *A. thaliana* [23]. Additionally, we showed that the same individual pure VOCs could suppress the formation of biofilms of three strains of *A. tumefaciens* of different origins and kill cells in mature biofilms [24].

**Figure 1:**
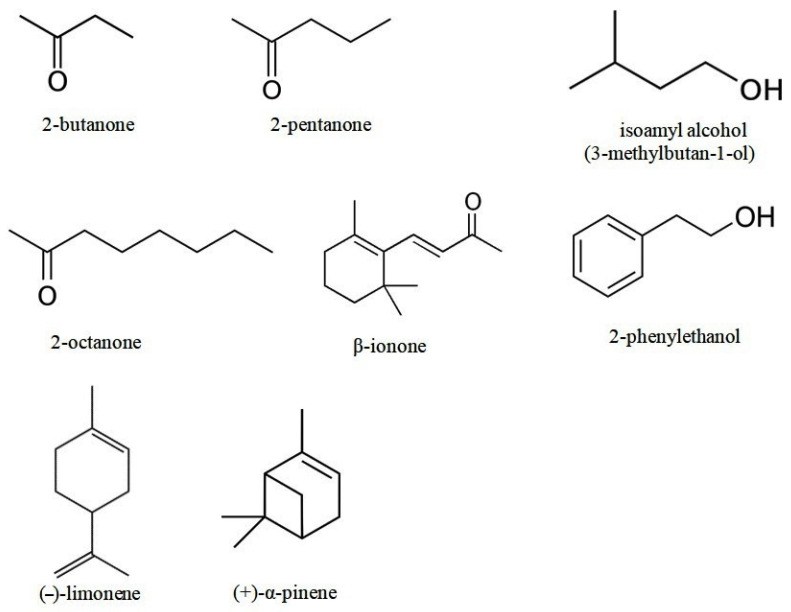
Volatile organic compounds used in this work.

In this work, several individual pure VOCs (Figure 1) belonging to three different chemical groups (ketones, alcohols, and terpenoids) and produced by various bacteria and fungi (according to the mVOC 2.0 database) were chosen for studying their biological activity. 

This study aimed to investigate the action of these compounds and to evaluate and compare their effect on various biological objects—agrobacteria, plants (*A. thaliana*, effect on plant growth and seed germination), and insects (*D. melanogaster*). New data obtained in this work are important for further study of these VOCs’ functional roles and mechanisms of action.

## 2. Materials and Methods

### 2.1. Organisms, Growth Conditions, and Chemicals

In this work, two *A. tumefaciens* strains were used: C58, nopaline type, isolated from cherry crown gall [25] and Chry5, chrysopine type, isolated from *Chrysanthemum* crown gall [26]. Bacteria were grown in liquid Luria–Bertani broth (LB) or on solid (1.5% *w/v* agar) Luria–Bertani agar (LA) medium (Sigma-Aldrich Chemie GmbH, Steinheim, Germany) at 28 °C [27].

The seeds of *A. thaliana* ecotype Columbia (accession CS70000; Col-0) were obtained from the ABRC Stock Center (https://abrc.osu.edu/stocks/number/CS70000, accessed on 21 December 2021). The plants were grown on agarized Murashige and Skoog (MS) Basal Medium plant cell culture with sucrose and agar (Sigma-Aldrich Chemie GmbH, Steinheim, Germany) at 24 °C.

*D. melanogaster* line F flies with the w1118 mutation (Drosophila Stock Center, Bloomington, IN, USA) were maintained at 24 °C on a yeast/sugar/raisins/agar medium containing 8 g of agar, 60 g of dried yeast, 40 g of sugar, 36 g of semolina, and 40 g of raisins, with water added to a 1-L final volume.

The pure VOCs of the following classes were studied (Figure 1): alcohols (2-phenylethanol and isoamyl alcohol; purity of both is >99%); ketones (2-butanone, 2-pentanone, 2-octanone, unsaturated ketone, and norterpenoid β-ionone; they all had >99% purity); and terpenes ((−)-limonene with >96% purity and (+)-α-pinene with >98% purity). All compounds were obtained from Sigma-Aldrich Chemie GmbH, Steinheim, Germany.

In each case, we tested the action of a wide range of VOC doses. We selected the doses of the VOCs, starting with those that had no effect and then increasing them to a level when they completely inhibited the subject’s vital functions (if it was possible). VOCs were taken directly from the initial liquid preparation without dilution in a solvent. 

### 2.2. The Action of VOCs on A. tumefaciens Growth

The effect of VOCs against *A. tumefaciens* strains was tested using a dual-culture assay as described [22]. Two-compartment plastic Petri plates (92 × 16 mm) were filled with LA medium. Fifty microliters of an overnight culture of *A. tumefaciens* strain grown in LB, diluted to about 10^6^ cells/mL, were placed on LA and distributed by a microbiological loop on the surface of the medium in one compartment of the plate. The chemical preparations of individual VOCs were placed on pieces of sterile filter paper (in an amount from several μL to ~200 μL) in the second compartment of the Petri plate. The plates were tightly sealed with four layers of Parafilm M (Pechiney Plastic Packaging Company, Chicago, IL, USA) to prevent VOC leakage and incubated at 28 °C. In the controls, the VOCs were omitted. Grown cells were harvested in physiological saline and plated from appropriate dilutions on LA medium. The results were analyzed after 2 days of bacterial growth at 28 °C. All experiments were repeated four times, with three plates per variant of the experiment.

### 2.3. Influence of VOCs on the Growth of A. thaliana Seedlings

Seeds of *A. thaliana* placed on filter paper in a Petri dish (92 × 16 mm) were sterilized with a solution of 5% H_2_O_2_ in 70% C_2_H_5_OH for 2 min. The seeds were then dried and transferred by a needle to a Petri dish with MS medium. The plates were incubated for 2 days at 4 °C. Then, the Petri dishes were removed from the refrigerator and set in a climate chamber in a 12-h light/12-h dark cycle at 24 °C. After 6 days, two cotyledonous leaves appeared. The seedlings were transferred into MS medium in one compartment of the Petri dish (3–5 seedlings in a dish). The tested VOCs were placed on strips of sterile filter paper in another dish compartment. The plates were tightly closed with 4 layers of Parafilm M and incubated in a climate chamber at 24 °C in a 12-h light/12-h dark cycle for two weeks. Finally, the plants were removed from the dishes, dried with sterile filter paper, and weighed on laboratory scales. Plants were grown under the conditions described above in the control plates, but VOCs were not added. All experiments were repeated four times, with three plates for one dose of the VOC.

### 2.4. Influence of VOCs on A. thaliana Seeds Germination

Sterilized *A. thaliana* seeds (see Section 2.3) were transferred by a microbiological loop to one compartment of the Petri dish filled with 7 mL of MS medium (20 seeds). The tested VOCs were placed on strips of sterile filter paper in another compartment of the Petri dish. The plates were tightly closed with 4 layers of Parafilm M and incubated in a climate chamber at 24 °C in a 12-h light/12-h dark cycle. The number of germinated seeds (seeds with root) and seeds with roots and two cotyledonous leaves was determined under a microscope Leica MZ6 (Leica Microsystems GmbH, Heerbrugg, Switzerland) and assayed on days 3, 6, and 9. In the control plates, VOCs were not added. All experiments were repeated four times, with three plates for one dose of the VOC.

### 2.5. Activity of VOCs against D. melanogaster

Ten flies (5 males and 5 females, 10 days of age) were transferred to a test tube (45 mL) containing agarized yeast/sugar/semolina/raisins medium. The tube was placed into a 340-mL glass container with a small foil box filled with a specified amount of VOC (in an amount from several μL to ~200 μL). The containers were tightly sealed with Parafilm M and incubated at 24 °C. The flies’ survival, growth, and development were analyzed on days 5, 9, and 12–14. In the control experiments, VOCs were omitted. The experiments were repeated four times with two test tubes containing 10 flies for each VOC dose.

### 2.6. Statistical Analysis

Statistical analysis of experiments was carried out using analysis software IBM SPSS software v. 26 (New York, NY, USA). The mean and standard errors were calculated using the Excel descriptive statistics program for the on-plate assays. Significant differences were determined by one-way ANOVA followed by Tukey’s HSD (Honestly Significant Difference) post hoc test. Differences were considered to be significant at *p ≤* 0.05.

## 3. Results

### 3.1. Effect of Individual Pure VOCs on A. tumefaciens Growth

The antibacterial effect of VOCs was investigated using *A. tumefaciens* strains C58 and Chry5 (Table 1).

Of the three volatile ketones studied, the most significant inhibition of bacterial growth was observed under the action of 2-octanone. The number of grown colonies of both strains of *A. tumefaciens* decreased at a dose of 15–50 μmol, and no growth was on the plates when 100 μmol of 2-octanone were added. The plates with 50 μmol of 2-octanone were unsealed to remove VOCs at the end of the experiments, and bacteria treated with 2-octanone slowly resumed their growth after 3–5 days. Inhibition of the *A. tumefaciens* growth under the action of 2-pentanone was found at higher amounts of ketone. At 100 μmol, the amount of CFU decreased ~ by half, and, at 200 μmol, four and nine times for strains C58 and Chry5, respectively, and with a further increase in the 2-pentanone amount, a sharp decrease in CFU was observed. 2-Butanone inhibited *A. tumefaciens* growth at significantly higher doses than 2-octanone. The growth of both strains was restored on the fifth day after plates were unsealed for removing 2-butanone or 2-pentanone. Hence, the tested ketones did not have a bactericidal effect on *A. tumefaciens* C58 and Chry5 cells under the conditions of experiments.

Of the alcohols studied, isoamyl alcohol exerted a more potent effect. The suppression of bacterial growth occurred already at 25 µmol and increased with an increase in the alcohol dose to 75 µmol; no visible cell growth was at 100 µmol. Bacterial growth was not restored within 6 days after the removal of isoamyl alcohol. 2-Phenylethanol had a significantly weaker effect on *Agrobacterium*. There was a gradual decrease in the CFU in the range of 200–600 μmol.

Studies of the action of two terpenes, (−)-limonene and (+)-α-pinene, showed that these compounds practically did not inhibit the growth of agrobacteria even at doses of 400 μmol ((−)-limonene) and 600 μmol ((+)-α-pinene). β-Ionone had little effect on agrobacteria at high doses; in the presence of β-ionone, the CFU/mL of the *A. tumefaciens* C58 and Chry5 strains decreased 1.8–2.9 times at a dose of 600 μmol, respectively.

### 3.2. Effect of VOCs on A. thaliana Growth

*A. thaliana* was used as a model plant in experiments on the effect of individual VOCs on plant growth (Table 2). It was shown that all the tested ketones could stimulate plant growth. Ketones 2-butanone (200–400 μmol) and 2-pentanone (200 μmol) increased the fresh weight of *A. thaliana* up to 1.5–2 times compared to the untreated control. 2-Octanone stimulated the plant growth at low doses: at 5 μmol up to 114% and at 10 up to 171%; with an increase in the amount of 2-octanone, plant growth was inhibited (Table 2), and the leaves and stems were discolored.

Isoamyl alcohol and 2-phenylethanol at 25 μmol and higher doses caused the inhibitory effect on *A. thaliana* growth. Under the action of high quantities of these compounds, some plants were totally white or almost transparent and very weak and thin. In the range of 50–200 µmol of alcohols, the plant biomass was lower than at 25 µmol and remained practically at the same level. Only doses 400 μmol and higher of β-ionone inhibited the plant’s growth. Under the action of (−)-limonene, the plant mass was only 45% of the untreated control at 200 and 400 μmol, and at 600 μmol, it sharply reduced to 3.5%. (+)-α-Pinene had no statistically significant effect on plant growth at doses up to 400 μmol.

### 3.3. Effect of VOCs on A. thaliana Seeds Germination

The analysis of the effect of VOCs on the seed germination of *A. thaliana* was carried out according to the method described by Lee et al. 2014 [28]. The results are presented in Table 3.

Of the three ketones studied, 2-octanone had a more substantial effect on seed germination. The formation of roots was inhibited on the third day of incubation at low amounts of this compound (5–40 μmol). Cotyledonous leaves did not appear on the third day when the seeds were incubated with these amounts of 2-octanone. However, after 6 and 9 days of incubation, the number of germinated seeds with leaves increased. 2-Pentanone, in an amount of 50 μmol after three days of incubation with seeds, reduced the root formation (2% seeds with roots compared to the untreated control) and completely inhibited the leaf growth; the effect increased with an increase in its amount to 100–200 μmol. 2-Butanone had a weak effect on seed germination and only at high doses (200 and 400 μmol).

On the ninth day, the complete suppression of seed germination was observed with 50 μmol and a higher amount of isoamyl alcohol. Compared to the untreated control, only 55% of the seeds germinated with 25 μmol of this compound on the third day, and cotyledonous leaves (28% of plants) appeared on day 6. Additionally, no roots and leaves appeared during the incubation of seeds with 25 μmol of 2-phenylethanol throughout the whole experiment, and only 73% of seeds gave leaves at a dose of 10 μmol of this substance on the ninth day. The effect of (−)-limonene was insignificant, and most of the seeds (83%) germinated at the highest amount of this compound (600 μmol) on the ninth day of the incubation. Cotyledonous leaves appeared on day 6 on almost all the germinated seeds. However, the leaves lost their green color to the end of the experiment. The effect of β-ionone was significantly stronger: after 3 days of incubation, the number of seeds with roots dropped sharply at 200–600 μmol of β-ionone in comparison with the control, and the leaves did not appear on the sixth and ninth days. The roots formed at these doses of β-ionone, although at a much smaller amount than in the control. Thus, seed root formation was inhibited by β-ionone to a lesser extent than leaf formation. (+)-α-Pinene practically did not affect the germination of the plant seeds.

### 3.4. Activity of VOCs against D. melanogaster

The results of the experiments on the effect of eight studied VOCs on *D. melanogaster* are shown in Table 4. In the control (without VOCs), after 12–14 days of the experiment, all the flies were alive; there were many larvae and pupae in the tubes, and new offspring appeared.

Of the tested ketones, 2-octanone had the most potent effect on *D. melanogaster*. This VOC killed all flies at 10 µmol after 12–14 days of incubation. Dead larvae and pupae were found at 10 and 15 µmol of 2-octanone after 9–14 days of incubation. At 25 and 50 µmol of 2-octanone, all the flies were dead already after 1 day of incubation, and larvae and pupae did not appear. 2-Pentanone also strongly affected the *D. melanogaster*, although its action was slightly weaker than that of 2-octanone. The third ketone, 2-butanone, acted weaker than the other two ketones. The unsaturated ketone β-ionone had a weak, delayed effect on *D. melanogaster*: at 100–400 µmol of β-ionone, after 9 days of incubation, all the flies were alive, and there were larvae and pupae, and only on the fourteenth day did all the flies, larvae, and pupae die. When a higher amount of β-ionone was used (600 and 800 µmol), all the flies were already dead by the ninth day. A small number of live larvae (but not pupae) were observed, but by the fourteenth day, they also died. Thus, the action of β-ionone slowed down the development of *D. melanogaster*, eventually leading to the deaths of the flies.

(−)-Limonene had a strong effect on *D. melanogaster* viability: at doses from 50 to 400 µmol, all the flies died after one day of incubation, and there were no larvae and pupae. At lower amounts of limonene, its effect was slower; at 10 and 25 µmol of (−)-limonene, all the flies died after 12–14 days and 9 days of incubation, respectively. The development of the flies was inhibited immediately after treatment with this VOC, and only a few larvae were observed in the tubes, already dead at the end of the incubation. Another member of the terpene group, (+)-α-pinene, acted somewhat weaker than (−)-limonene. At 10 µmol of (+)-α-pinene after 5 days of incubation, all the flies were alive and active; larvae and pupae appeared at 9 days, but they were all dead after 12–14 days of treatment. At 25 µmol of (+)-α-pinene, after 9 days of incubation, there were only a small number of dead larvae and no pupae, i.e., at this amount of (+)-α-pinene, the development of *Drosophila* was sharply inhibited. With a further increase of the (+)-α-pinene amount, practically all the flies were dead, and no larvae were observed during the entire incubation period.

The investigated alcohols (2-phenylethanol and isoamyl alcohol) had a weak effect on *Drosophila.* At 50 and 100 µmol of 2-phenylethanol, all the flies were alive and actively multiplying; there were many larvae and pupae. At doses of 200–600 µmol, all the flies died after only 14 days; after 9 days, there were many live larvae and pupae, but by the fourteenth day, they were dead. Isoamyl alcohol began to kill flies at a dose of 100 µmol; the number of dead flies increased with increasing the incubation time; all larvae died after 14 days. At quantities of isoamyl alcohol of 200 and 400 µmol, all the flies died after incubation for 5 days with this alcohol; a large number of flies were dead after 1 day of incubation. During the entire incubation period, no larvae were observed; that is, the development of *Drosophila* was immediately stopped by the action of these doses of isoamyl alcohol.

At the end of the experiments, we removed the VOC vials from the containers where the flies and larvae were immobile and apparently dead to test whether the VOCs do indeed have an insecticidal effect. In all cases, we did not observe the recovery of the vital activity of the flies and larvae. 

## 4. Discussion

Historically, VOCs emitted by microorganisms began to be studied later than VOCs of plants, but they have received increasing attention over the last decade. Nowadays, the potential biotechnological application of VOCs is considered in agriculture, medicine, the food industry, and many other important fields; they can be used as total pools of volatile products emitted by bacterial strains and as individual pure volatile substances [1,12,16].

This work studied the effect of several pure chemically synthesized VOCs with diverse structures. These VOCs are emitted by bacteria of different taxonomic groups [8]. We showed the action of VOCs on phytopathogenic Gram-negative bacteria *A. tumefaciens* (strains C58 and Chry5), the growth and germination of seeds of *A. thaliana,* and the viability of fruit fly *D. melanogaster*.

Of the six ketones (2-butanone, 2-pentanone, 2-heptanone, 2-octanone, 2-nonanone, and 2-undecanone) studied in this and previous works [22], 2-nonanone, 2-heptanone, and 2-octanone exhibited the most substantial inhibiting effect on *A. tumefaciens*. The impact of 2-pentanone was weaker than the other three ketones and that of 2-butanone was even weaker. Comparison of the action of these ketones on the growth of agrobacteria suggested that the effectiveness of ketones, presumably, correlates with the length of their hydrocarbon chain (see Figure 1). The only exception to this pattern was 2-undecanone (11C), which had a weaker effect on agrobacteria [22].

The activity of 2-methylketones, like 2-nonanone, was already described previously and was dependent on the lipophilicity and, thus, chain length of the ketones determining their ability to cross lipid barriers of cell membranes. For the insecticide 2-tridecanone and its analogs, it was shown that an alkyl chain length of 9–14 carbon atoms seemed to be most effective against the tomato fruitworm (*Heliothis zea*) [29].

It can be assumed that the inhibitory activity of the ketones indicated is associated with the ability of these substances to interact with hydrophobic segments in proteins and to denature proteins [30,31]. The hydrophobic nature of the interaction of ketone 2-nonanone with protein bovine serum albumin was demonstrated by physical methods [30]. None of the studied VOCs promoted the growth of agrobacteria.

The ability of VOCs to promote plant growth is currently of great interest. This effect was first discovered in 2003 by Ryu and coworkers [32]. It has been shown that 2,3-butanediol promotes the growth of *A. thaliana* and induces the systemic resistance of plants [32,33]. Afterwards, it was found that other VOCs of various chemical nature emitted by bacteria have promoted the growth of plants, i.e., 2-pentylfurane, indole, pentadecane, 1-hexanol, dimethyl disulfide, and acetophenone (*A. thaliana*); dimethylhexadecylamine (*Medicago sativa*); and 3-hydroxy-2-butanone (*Nicotianatabacum*) [7,15,34,35,36,37]. The number of newly discovered VOCs, plant growth inducers, is increasing.

In this work, we showed that ketones 2-butanone and 2-pentanone in relatively high amounts increased the fresh weight of *A. thaliana*. 2-Octanone stimulated the plant growth at low doses; an increase in its quantity led to plant growth inhibition. Our results on the 2-butanone effect on *A. thaliana* correlate with those received earlier about the beneficial actions of 2-butanone on tobacco plants [38]. The alcohols isoamyl alcohol and 2-phenylethanol and terpene (−)-limonene had an inhibitory effect on the growth of *A. thaliana* seedlings.

The question of the mechanisms of the stimulating effect of VOCs on plant growth is of great interest, but it has not been sufficiently studied yet. VOCs are supposed to promote plant growth by increasing photosynthesis and sugar accumulation in plants and modulating phytohormone signaling; additionally, they can improve the uptake of minerals [36]. The mechanism of *A. thaliana* growth promotion by 2-butanone, 2-pentanone, and 2-octanone is unclear. Isoamyl alcohol and 2-phenylethanol at an amount of 25 μmol and higher caused the inhibitory effect of the vital activity of plants. β-Ionone and (−)-limonene decreased the plant biomass at high amounts (400–600 μmol). None of the VOCs used stimulated *A. thaliana* seed germination.

Insects have complex chemosensory systems that are very sensitive to volatile chemical signals [39]. It has been shown that some VOCs formed mainly by fungi (2-octanone, 3-octanol, and 2–5-dimethylfuran) lead to the death of *D. melanogaster*. These VOCs are neurotoxic. They cause truncated lifespans, locomotory defects, and changes in dopaminergic neurons in adult *D. melanogaster*. Their action is suggested to be associated with the synthesis of reactive oxygen species (ROS). ROS cause lipid peroxidation-inducing effects, leading to the generation of toxic products. The increase of lipid peroxidation production via the generation of ROS may be associated with the toxicity of these VOCs [40,41,42].

The sensitivity of different organisms to the VOCs we studied can be different. For example, (−)-limonene has the most substantial effect (at lower doses) on *D. melanogaster* and almost no effect on the growth of *A. thaliana*, the germination of seeds of this plant, and viability of *A. tumefaciens*. It should be taken into account that the added amount of this VOC falls on a larger volume of the vessel (340-mL glass container) in the case of experiments with flies compared to the volume of Petri dishes (~90 mL), in which experiments with bacteria and plants were carried out. Another terpene, (+)-α-pinene, acts on *D. melanogaster* similarly to (−)-limonene and practically does not act on plants and bacteria. There are differences in the sensitivity of various organisms to other VOCs, which is not surprising, since the effects of VOCs have been studied on organisms belonging to different kingdoms. The targets of VOC actions in these organisms could be different.

Obtained data open new points for discussions about VOCs, mechanisms of their actions, and the roles of these compounds in the relationships of microorganisms and their interactions with higher organisms.

## 5. Conclusions

Studying volatile substances of microorganisms, their functional role, and biological activities is currently of great interest to researchers working in various fields of fundamental and applied biology. The chemical diversity of VOCs released by microorganisms provides a source of new substances that can be used in medicine, biotechnology, and agriculture. Based on VOCs, new types of pesticides are being developed that do not harm the environment. Bacterial strains that synthesize gas mixtures of volatile substances and individual pure VOCs can be used against phytopathogenic microorganisms—bacteria, fungi, and other plant pests. Pure VOCs showed promising results in improving plant growth and suppressing pests and diseases in the field [43]. For the successful use of VOCs, it is necessary to know the mechanisms of their actions, which have been little studied, and patterns of actions of VOCs on various organisms living in natural conditions. It is appropriate to investigate these questions primarily for individual pure VOCs. Understanding those will be a prerequisite for developing strategies for applying VOCs in agriculture and other fields in the future. 

In this work, we showed that pure individual VOCs of various chemical structure act on organisms belonging to distant taxonomic groups (phytopathogenic agrobacteria, plants *A. thaliana*, and fruit flies *D. melanogaster*) that can live in the same ecological niche. The effect of VOCs can be different—from inhibiting growth and killing the organism to promoting the growth of plants; the types of actions of VOCs depend on the target organism. Our data can be useful for the development of new methods of plant protection against phytopathogens and the fumigation of soils in agriculture using individual VOCs and bacteria producers of volatile compounds. 

## Figures and Tables

**Table 1:** Effect of VOCs on *A. tumefaciens* strain growth.

Amount of VOCs, μmol	Quantity of Colony-Forming Units of *A. tumefaciens* Strains (CFU/mL)	
	C58	Chry5
	2-butanone	
0 (Control)	(1.2 ± 0.6 × 10^10^) ^a^	(4 ± 1.1 × 10^10^) ^a^
100	(1.3 ± 0.4 × 10^10^) ^a^	(2.4 ± 0.8 × 10^10^) ^b^
200	(0.98 ± 3 × 10^10^) ^a^	(1.9 ± 0.8 × 10^10^) ^b^
400	(5.5 ± 2.1 × 10^8^) ^b^	(5.5 ± 1.7 × 10^7^) ^c^
	2-pentanone	
0 (Control)	(5.9 ± 0.8 × 10^10^) ^a^	(4.8 ± 0.6 × 10^10^) ^a^
100	(3.2 ± 0.5 × 10^10^) ^b^	(2.3 ± 0.6 × 10^10^) ^b^
200	(1.4 ± 0.2 × 10 ^10^) ^c^	(5.1 ± 1.3 × 10^9^) ^c^
400	(1.9 ± 0.7 × 10 ^9^) ^d^	(1.7 ± 1.1 × 10^7^) ^c^
	2-octanone	
0 (Control)	(6.3 ± 1.2 × 10^10^) ^a^	(8.5 ± 3.5 × 10^10^) ^a^
15	(2.2 ± 0.5 × 10^10^) ^b^	(5.2 ± 0.4 × 10^10^) ^a,b^
25	(1.8 ± 0.6 × 10^10^) ^b,c^	(3.2 ± 0.8 × 10^10^) ^b,c^
50	(2.3 ± 0.7 × 10^9^) ^c^	(7.7 ± 1 × 10^9^) ^c^
100	* ng	ng
	β-ionone	
0 (Control)	(6.1 ± 2 × 10^10^) ^a^	(7.2 ± 2.3 × 10^10^) ^a^
200	(5.6 ± 1.2 × 10^10^) ^a^	(6.1 ± 1.4 × 10^10^) ^a,b^
400	(4.1 ± 0.8 × 10^10^) ^a,b^	(3.8 ± 1.4 × 10^10^) ^a,b^
600	(3.4 ± 1.1 × 10^10^) ^a,b^	(2.5 ± 0.8 × 10^10^) ^b^
800	(1.1 ± 0.3 × 10^10^) ^b^	-
	isoamyl alcohol	
0 (Control)	(4.1 ± 1 × 10^10^) ^a^	(5.4 ± 1.7 × 10^10^) ^a^
25	(2.7 ± 0.6 × 10^10^) ^a,b^	(2.4 ± 0.7 × 10^10^) ^b^
50	(1.3 ± 0.4 × 10^10^) ^b^	(1.3 ± 0.4 × 10^10^) ^b^
75	(0.73 ± 1.2 × 10^9^) ^b^	(7.5 ± 0.9 × 10^9^) ^b^
100	ng *	ng
	2-phenylethanol	
0 (Control)	(1.88 ± 0.2 × 10^10^) ^a^	(4.5 ± 0.8 × 10^10^) ^a^
200	(2.6 ± 0.1 × 10^9^) ^b^	(5.4 ± 0.5 × 10^9^) ^b^
400	(2.8 ± 0.2 × 10^9^) ^b^	(4.1 ± 1 × 10^9^) ^b^
600	(1.1 ± 0.6 × 10^9^) ^b^	(2.9 ± 0.4 × 10^9^) ^b^
	(−)-limonene	
0 (Control)	(3.5 ± 1 × 10^10^) ^a^	(2.7 ± 1.1 × 10^10^) ^a^
200	(3.4 ± 0.4 × 10^10^) ^a,b^	(1.8 ± 0.5 × 10^10^) ^a^
400	(3.3 ± 0.7 × 10^10^) ^a,b^	(2.35 ± 1 × 10^10^) ^a^
600	(1.6 ± 0.6 × 10^10^) ^b^	(1.95 ± 0.9 × 10^10^) ^a^
	(+)-α-pinene	
0 (Control)	(5.7 ± 1.1 × 10^10^) ^a^	(12 ± 2.3 × 10^10^) ^a^
200	(5.7 ± 0.4 × 10^10^) ^a^	(14.6 ± 2.7 × 10^10^) ^a^
400	(5.8 ± 0.3 × 10^10^) ^a^	(13.4 ± 1.3 × 10^10^) ^a^
600	(5.6 ± 1.8 × 10^10^) ^a^	(13.3 ± 2.5 × 10^10^) ^a^

* ng: no visible growth; For each strain and VOC, the different lowercase letters above the means indicate significant differences (*p* ≤ 0.05; Tukey’s HSD test).

**Table 2:** Effect of VOCs on *A. thaliana* biomass *.

VOC	Amount of VOCs, μmol/Plant Biomass in % of Control				
2-butanone	50	100	200	400	
	(102.3 ± 15.5) ^a^	(95 ± 14.6) ^a^	(146.1 ± 13) ^b^	(195.6 ± 27.8) ^c^	-
2-pentanone	50	100	200		
	(93.34 ± 13.4) ^a^	(129.5 ± 19.7) ^a^	(152.2 ± 22.3) ^b^	-	-
2-octanone	5	10	20	30	40
	(114.6 ± 17.1) ^a^	(171.1 ± 26.6) ^b^	(77.8 ± 26.4) ^c^	(21.6 ± 8.6) ^d^	(13.9 ± 6.2) ^d^
β-ionone	200	400	600		
	(94 ± 20.6) ^b^	(22.5 ± 8.3) ^b,c^	(11.06 ± 6.1) ^c^	-	-
isoamyl alcohol	25	50	75	100	
	(45.08 ± 18.3) ^b^	(23.1 ± 14.4) ^b,c^	(20.1 ± 15.1) ^c^	(29.1 ± 11.7) ^b,c^	-
2-phenylethanol	25	50	100	200	
	(60.4 ± 20.2) ^a^	(21.9 ± 16.1) ^b^	(22.14 ± 12.1) ^b^	(27.6 ± 9.3) ^b^	-
(−)-limonene	200	400	600		
	(45.4 ± 16.2) ^b^	(44.6 ± 6.7) ^b^	(3.5 ± 2.6) ^c^	-	-
(+)-α-pinene	100	200	400		
	(90 ± 12.3) ^a^	(81 ± 18.8) ^a^	(94.2 ± 20.4) ^a^	-	-

* Plant biomass (fresh weight) is shown as a percentage of the control (no VOC was added). 100 ^a^%—plant biomass in the control. For each VOC, the different lowercase letters above the means indicate significant differences (*p* ≤ 0.05; Tukey’s HSD test).

**Table 3:** Effect of VOCs on the germination of *A. thaliana* seeds.

VOC	Amount of VOCs, µmol	3rd Day		6th Day		9th Day	
		1 *	2 **	1	2	1	2
2-butanone	0	(93 ± 5) ^a^	(58 ± 9) ^a^	(98 ± 2) ^a^	(98 ± 2) ^a^	100 ^a^	100 ^a^
	50	(93 ± 6) ^a^	(41 ± 5) ^a^	(98 ± 2) ^a^	(98 ± 2) ^a^	100 ^a^	100 ^a^
	100	100 ^a^	0 ^b^	100 ^a^	100 ^a^	100 ^a^	100 ^a^
	200	(72 ± 3) ^a^	0 ^b^	100 ^a^	(97 ± 2) ^a^	100 ^a^	100 ^a^
	400	(2 ± 0.2) ^b^	0 ^b^	(93 ± 6) ^a^	(35 ± 1) ^b^	(93 ± 4) ^a^	(92 ± 3) ^b^
2-pentanone	0	(83 ± 3) ^a^	(74 ± 6) ^a^	(93 ± 6) ^a,b^	(93 ± 6) ^a^	100 ^a^	(93 ± 6) ^a^
	50	(2 ± 1) ^b^	0 ^b^	(97 ± 3) ^a^	(80 ± 7) ^a^	(98 ± 1) ^a^	(98 ± 1) ^a^
	100	0 ^b^	0 ^b^	(78 ± 5) ^b^	(18 ± 1) ^b^	(95 ± 1) ^a^	(70 ± 6) ^a^
	200	0 ^b^	0 ^b^	0 ^c^	0 ^b^	(8 ± 5) ^b^	0 ^b^
2-octanone	0	(98 ± 2) ^a^	(85 ± 5) ^a^	100 ^a^	100 ^a^	100 ^a^	100 ^a^
	5	(78 ± 3) ^a,b^	0 ^b^	(95 ± 3) ^a^	(83 ± 7) ^a^	(98 ± 2) ^a^	(97 ± 3) ^a^
	10	(90 ± 5) ^a^	0 ^b^	(100) ^a^	(87 ± 12) ^a^	(100) ^a^	100 ^a^
	20	(45 ± 1) ^b,c^	0 ^b^	(97 ± 3) ^a^	(22 ± 16) ^b^	(98 ± 2) ^ab^	(98 ± 2) ^a^
	30	(18 ± 1) ^c^	0 ^b^	(82 ± 1) ^a^	(15 ± 1) ^b^	(93 ± 3) ^ab^	(82 ± 8) ^a,b^
	40	(15 ± 1) ^c^	0 ^b^	(40 ± 15) ^b^	0 ^b^	(78 ± 15) ^b^	(50 ± 18) ^b^
β-ionone	0	(92.2 ± 5) ^a^	(32 ± 8) ^a^	(97 ± 3) ^a^	(97 ± 3) ^a^	100 ^a^	100 ^a^
	25	(95 ± 5) ^a^	0 ^b^	(98 ± 2) ^a^	(98 ± 2) ^a^	100 ^a^	100 ^a^
	50	(90 ± 1) ^a^	0 ^b^	(95 ± 5) ^a^	(85 ± 9) ^a^	(95 ± 3) ^a^	(95 ± 3) ^a^
	100	(85 ± 1) ^a^	0 ^b^	(96 ± 4) ^a^	(52 ± 10) ^b^	(95 ± 2) ^a^	(58 ± 10) ^b^
	200	(8 ± 3) ^b^	0 ^b^	(17 ± 8) ^b^	0 ^c^	(33 ± 13) ^b^	0 ^c^
	400	(12 ± 3) ^b^	0 ^b^	(12 ± 3) ^b^	0 ^c^	(15 ± 5) ^c^	0 ^c^
	600	(3 ± 2) ^b^	0 ^b^	(4 ± 3) ^b^	0 ^c^	(7 ± 3) ^c^	0 ^c^
isoamyl alcohol	0	(98 ± 2) ^a^	(92 ± 5) ^a^	(98 ± 2) ^a^	(98 ± 2) ^a^	100 ^a^	100 ^a^
	10	(97 ± 3) ^a^	(3 ± 2) ^b^	100 ^a^	(95 ± 5) ^a^	100 ^a^	100 ^a^
	25	(55 ± 13) ^b^	0 ^b^	(51 ± 2) ^b^	(28 ± 3) ^b^	(99 ± 1) ^a^	(83 ± 1) ^b^
	50	0 ^c^	0 ^b^	0 ^c^	0 ^c^	0 ^b^	0 ^c^
	75	0 ^c^	0 ^b^	0 ^c^	0 ^c^	0 ^b^	0 ^c^
	100	0 ^c^	0 ^b^	0 ^c^	0 ^c^	0 ^b^	0 ^c^
2-phenylethanol	0	(97 ± 2) ^a^	(80 ± 2) ^a^	(98 ± 2) ^a^	(98 ± 2) ^a^	100 ^a^	100 ^a^
	5	(90 ± 3) ^a^	(7 ± 3) ^b^	(98 ± 2) ^a^	(68 ± 5) ^a^	(98 ± 2) ^a^	(95 ± 5) ^a^
	10	(52 ± 7) ^b^	0 ^b^	(90 ± 2) ^b^	(10 ± 1) ^b^	(95 ± 3) ^a^	(73 ± 2) ^b^
	25	0 ^c^	0 ^b^	0 ^c^	0 ^c^	0 ^b^	0 ^c^
	50	0 ^c^	0 ^b^	0 ^c^	0 ^c^	0 ^b^	0 ^c^
	100	0 ^c^	0 ^b^	0 ^c^	0 ^c^	0 ^b^	0 ^c^
	200	0 ^c^	0 ^b^	0 ^c^	0 ^c^	0 ^b^	0 ^c^
(−)-limonene	0	(83 ± 5) ^a^	(74 ± 6) ^a^	(93 ± 6) ^a^	(93 ± 6) ^a^	100 ^a^	(93 ± 6) ^a^
	200	(47 ± 7) ^a^	0 ^b^	(93 ± 2) ^a^	(93 ± 2) ^a^	(93 ± 5) ^a^	(93 ± 5) ^a^
	400	(62 ± 1) ^a^	0 ^b^	(96 ± 4) ^a^	(93 ± 7) ^a^	(98 ± 2) ^a^	(93 ± 6) ^a^
	600	(42 ± 3) ^a^	0 ^b^	(80 ± 8) ^a^	(77 ± 8) ^a^	(83 ± 6) ^a^	(83 ± 6) ^a^
(+)-α-pinene	0	(97 ± 3) ^a^	(97 ± 3) ^a^	(97 ± 3) ^a^	(97 ± 3) ^a^	(97 ± 3) ^a^	(97 ± 3) ^a^
	100	(97 ± 3) ^a^	(97 ± 3) ^a^	(97 ± 3) ^a^	(97 ± 3) ^a^	(98 ± 2) ^a^	(98 ± 2) ^a^
	200	(97 ± 3) ^a^	(85 ± 10) ^a^	(98 ± 5) ^a^	(95 ± 5) ^a^	(98 ± 2) ^a^	(98 ± 2) ^a^
	400	(88 ± 4) ^a^	(77 ± 10) ^b^	(90 ± 5) ^a^	(90 ± 5) ^a^	(90 ± 5) ^a^	(90 ± 5) ^a^

* 1—seed germination (seed with a root in % of the total number of seeds) and ** 2—the appearance of two cotyledonous leaves (in % of the total number of seeds). For each time point and VOC, the different lowercase letters above the means indicate significant differences (*p* ≤ 0.05; Tukey’s HSD test).

**Table 4:** Effect of VOCs on *Drosophila melanogaster*.

Amount of VOCs, μmol	The Number of Dead *Drosophila* Flies *			Amount of VOCs, μmol	The Number of Dead *Drosophila* Flies *		
	5 Days	9 Days	12–14 Days		5 Days	9 Days	12–14 Days
2-butanone				isoamyl alcohol			
50	(2 ± 1) ^a^	(10 ± 0) ^b^	(10 ± 0) ^b^	25	0 ^a^	0 ^a^	0 ^a^
100	(3 ± 2) ^a,b^	(10 ± 0) ^b^	(10 ± 0) ^b^	50	0 ^a^	0 ^a^	0 ^a^
200	(6 ± 2) ^b^	(10 ± 0) ^b^	(10 ± 0) ^b^	100	(1 ± 1) ^b^	(4 ± 4) ^b^	(6 ± 3) ^b^
300	(10 ± 0) ^c^	(10 ± 0) ^b^	(10 ± 0) ^b^	200	(10 ± 0) ^c^	(10 ± 0) ^c^	(10 ± 0) ^c^
2-pentanone				400	(10 ± 0) ^c^	(10 ± 0) ^c^	(10 ± 0) ^c^
10	0 ^a^	(2 ± 1) ^b^	(10 ± 0) ^b^	2-phenylethanol			
25	(2 ± 2) ^a^	(10 ± 0) ^c^	(10 ± 0) ^b^	50	0 ^a^	0 ^a^	0 ^a^
50	(9 ± 1) ^b^	(10 ± 0) ^c^	(10 ± 0) ^b^	100	0 ^a^	0 ^a^	0 ^a^
100	(10 ± 0) ^b^	(10 ± 0) ^c^	(10 ± 0) ^b^	200	0 ^a^	0 ^a^	(10 ± 0) ^b^
2-octanone				400	0 ^a^	0 ^a^	(10 ± 0) ^b^
10	(1 ± 1) ^a^	(6 ± 2) ^b^	(10 ± 0) ^b^	600	0 ^a^	0 ^a^	(10 ± 0) ^b^
15	(6 ± 2) ^b^	(9 ± 1) ^c^	(10 ± 0) ^b^	(−)-limonene			
25	(10 ± 0) ^c^	(10 ± 0) ^c^	(10 ± 0) ^b^	10	(1 ± 0) ^a^	(4 ± 0) ^b^	(10 ± 0) ^b^
50	(10 ± 0) ^c^	(10 ± 0) ^c^	(10 ± 0) ^b^	25	(3 ± 1) ^b^	(10 ± 0) ^c^	(10 ± 0) ^b^
β-ionone				50	(10 ± 0) ^c^	(10 ± 0) ^c^	(10 ± 0) ^b^
100	0 ^a^	0 ^a^	(10 ± 0) ^b^	100	(10 ± 0) ^c^	(10 ± 0) ^c^	(10 ± 0) ^b^
200	0 ^a^	0 ^a^	(10 ± 0) ^b^	200	(10 ± 0) ^c^	(10 ± 0) ^c^	(10 ± 0) ^b^
300	0 ^a^	0 ^a^	(10 ± 0) ^b^	400	(10 ± 0) ^c^	(10 ± 0) ^c^	(10 ± 0) ^b^
400	0 ^a^	0 ^a^	(10 ± 0) ^b^	(+)-α-pinene			
600	0 ^a^	(10 ± 0) ^b^	(10 ± 0) ^b^	10	0 ^a^	(1 ± 1) ^b^	(5 ± 1) ^b^
				25	(5 ± 2) ^b^	(10 ± 0) ^c^	(10 ± 0) ^c^
800	0 ^a^	(10 ± 0) ^b^	(10 ± 0) ^b^	50	(9 ± 1) ^c^	(10 ± 0) ^c^	(10 ± 0) ^c^
				100	(10 ± 0) ^c^	(10 ± 0) ^c^	(10 ± 0) ^c^
				200	(10 ± 0) ^c^	(10 ± 0) ^c^	(10 ± 0) ^c^
				400	(10 ± 0) ^c^	(10 ± 0) ^c^	(10 ± 0) ^c^

* The number of flies dead was calculated per tube (of 10 flies). The number of dead flies in the control is 0 ^a^ at the indicated time point. For each time point and VOC, the different lowercase letters above the means indicate significant differences (*p* ≤ 0.05; Tukey’s HSD test).

## Data Availability

The author elects not to share data.
